# Overexpression of the trehalose-6-phosphate phosphatase family gene *AtTPPF* improves the drought tolerance of *Arabidopsis thaliana*

**DOI:** 10.1186/s12870-019-1986-5

**Published:** 2019-09-02

**Authors:** Qingfang Lin, Jiao Yang, Qiongli Wang, Hong Zhu, Zhiyong Chen, Yihang Dao, Kai Wang

**Affiliations:** 1 Key Laboratory of Genetics, Breeding and Multiple Utilization of Crops, Ministry of Education, Fujian Provincial Key Laboratory of Haixia Applied Plant Systems Biology, Center for Genomics and Biotechnology, Fujian Agriculture and Forestry University, Fuzhou, 350002 Fujian China; 2 National Engineering Research Center of Sugarcane, Fujian Agriculture and Forestry University, Fuzhou, 350002 China

**Keywords:** *Arabidopsis thaliana*, *AtTPPF*, DREB1A, Trehalose, SAM, Drought tolerance

## Abstract

**Background:**

Trehalose-6-phosphate phosphatases (TPPs), which are encoded by members of the *TPP* gene family, can improve the drought tolerance of plants. However, the molecular mechanisms underlying the dynamic regulation of *TPP* genes during drought stress remain unclear. In this study, we explored the function of an *Arabidopsis TPP* gene by conducting comparative analyses of a loss-of-function mutant and overexpression lines.

**Results:**

The loss-of-function mutation of *Arabidopsis thaliana TPPF*, a member of the *TPP* gene family, resulted in a drought-sensitive phenotype, while a line overexpressing *TPPF* showed significantly increased drought tolerance and trehalose accumulation. Compared with wild-type plants, *tppf1* mutants accumulated more H_2_O_2_ under drought, while *AtTPPF*-overexpressing plants accumulated less H_2_O_2_ under drought. Overexpression of *AtTPPF* led to increased contents of trehalose, sucrose, and total soluble sugars under drought conditions; these compounds may play a role in scavenging reactive oxygen species. Yeast one-hybrid and luciferase activity assays revealed that DREB1A could bind to the DRE/CRT element within the *AtTPPF* promoter and activate the expression of *AtTPPF*. A transcriptome analysis of the *TPPF*-overexpressing plants revealed that the expression levels of drought-repressed genes involved in electron transport activity and cell wall modification were upregulated, while those of stress-related transcription factors related to water deprivation were downregulated. These results indicate that, as well as its involvement in regulating trehalose and soluble sugars, *AtTPPF* is involved in regulating the transcription of stress-responsive genes.

**Conclusion:**

*AtTPPF* functions in regulating levels of trehalose, reactive oxygen species, and sucrose levels during drought stress, and the expression of *AtTPPF* is activated by DREB1A in *Arabidopsis.* These findings shed light on the molecular mechanism by which *AtTPPF* regulates the response to drought stress.

**Electronic supplementary material:**

The online version of this article (10.1186/s12870-019-1986-5) contains supplementary material, which is available to authorized users.

## Background

Trehalose is a non-reducing disaccharide comprising two Glc units linked in an α, α-1, 1-glucoside configuration. This compound exists in plants, fungi, bacteria, and invertebrate animals [1]. In addition to its function as a carbon source, it also acts as a protective compound under adverse conditions, such as dehydration, high salinity, hypoxia, and nutrient starvation [1, 2]. For example, trehalose functions as a protectant to stabilize membranes and proteins in certain resurrection plants such as *Myrothamnus flabellifolius* and *Sporobolus* spp., allowing them to survive during dehydration–rehydration cycles [3, 4].

Five trehalose synthesis pathways exist in prokaryotes, but only the trehalose-6-phosphate (T6P) synthase (TPS)/T6P phosphatase (TPP) pathway exists in eukaryotes [1]. In this pathway, T6P is first synthesized from UDP-Glc and Glc-6-phosphate by TPS and then dephosphorylated to trehalose by TPP [1]. As an important phosphorylated intermediate in the trehalose synthetic pathway, T6P is an essential sugar-signaling metabolite that regulates plant metabolism and other biological processes [5–8]. The molecular mechanisms underlying the accumulation of trehalose and T6P, with the latter contributing more to increased drought tolerance, are not yet understood. However, the accumulation of soluble sugars such as sucrose and trehalose may be a protective mechanism under oxidative stress conditions [9–11]. Because of the relatively low abundance of trehalose, it may have little effect on osmotic regulation unless its distribution within cells is specifically compartmentalized [12]. In *Arabidopsis*, T6P is a specific signaling molecule that senses the sucrose concentration [13, 14]. Because T6P exists at a concentration three orders of magnitude lower than that of sucrose, it is possible that a slight change in the T6P level may be accompanied by dramatic changes in the sucrose concentration [13, 14]. Therefore, changes in T6P levels may contribute more than changes in other sugars to increasing stress tolerance.

The constitutive heterologous overexpression of trehalose biosynthetic genes is a valuable tool for improving the stress tolerance of plants. Thus, *TPS* and *TPP* genes have received considerable attention because of their important roles in trehalose biosynthesis [15, 16]. The genome of *Arabidopsis thaliana* contains 11 *TPS*s (*AtTPS1*–*11*) and 10 *TPP*s (*AtTPPA–J*) [16]. Of them, AtTPS1, − 2 and − 4 -exhibit TPS enzymatic activity and all of the TPP proteins exhibit TPP enzymatic activity when heterologously expressed in yeast [15–17]. The TPSs and TPPs function as important growth regulators during responses to adverse conditions and in the formation of crop yield. The overexpression of *AtTPS1* conferred dehydration tolerance in *Arabidopsis*, accompanied by small increases in trehalose and T6P levels [18]. The wheat *TPP* gene *TPP-6AL1* was found to be associated with 1000-grain weight and grain yield [19]. From a practical standpoint, the overexpression of rice *TPP1* in maize under the control of the flower-specific promoter *MADS6* improved yield under varying degrees of drought [20]. Much of the research on trehalose biosynthetic genes has focused on their role of trehalose in stress responses and their function in increasing crop production. The underlying mechanism of *TPP* regulation and the dynamic regulation of related genes under drought conditions are still unclear.

The 10 *TPP* genes in *Arabidopsis* exhibit diverse spatiotemporal expression patterns, indicating that they may have distinct functions [16]. *AtTPPD* is preferentially expressed in the root cap, and the overexpression of *AtTPPD* can improve salt tolerance, indicating that this gene plays a role in the response to saline conditions [12]. *AtTPPA* and *AtTPPG* tend to be expressed in the protoderm and may play redundant roles during the differentiation of root epidermal cells [21]. *AtTPPB* is highly expressed in young leaves and functions in increasing the number of leaf cells [21]. *AtTPPG* has a particular expression profile in stomatal cells and is involved in the regulation of stomatal closure, with *tppg* mutants exhibiting resistance to abscisic acid (ABA)-mediated stomatal closure [21]. Interestingly, *AtTPPF* is induced specifically under dehydration conditions. Additionally, compared with wild-type (WT) plants, *AtTPPF*-overexpressing plants were found to be slightly more sensitive to ABA treatments, as assessed by a stomatal movement assay [16, 22]. This suggests that *AtTPPF* plays an important role in regulating stomatal movement and may be involved in the regulation of drought responses. Although rice (*Oryza sativa*) *TPP1* was shown to improve tolerance to dehydration and improve grain yields in maize [20], the molecular mechanisms by which TPP regulates drought responses remain largely unknown.

Here, we provide evidence that the loss-of-function mutant of *AtTPPF* results in a drought-sensitive phenotype and that the elevated expression of *AtTPPF* can increase drought tolerance. We discovered that DREB1A targets *AtTPPF* and mediates drought stress signaling by directly binding the DRE/CRT motif in the *AtTPPF* promoter, thereby activating its transcription. A global transcriptional analysis revealed that *AtTPPF* functions in regulating the transcription of genes involved in stress responses.

## Results

### *tppf1* mutant is drought sensitive

**Fig. 1:**
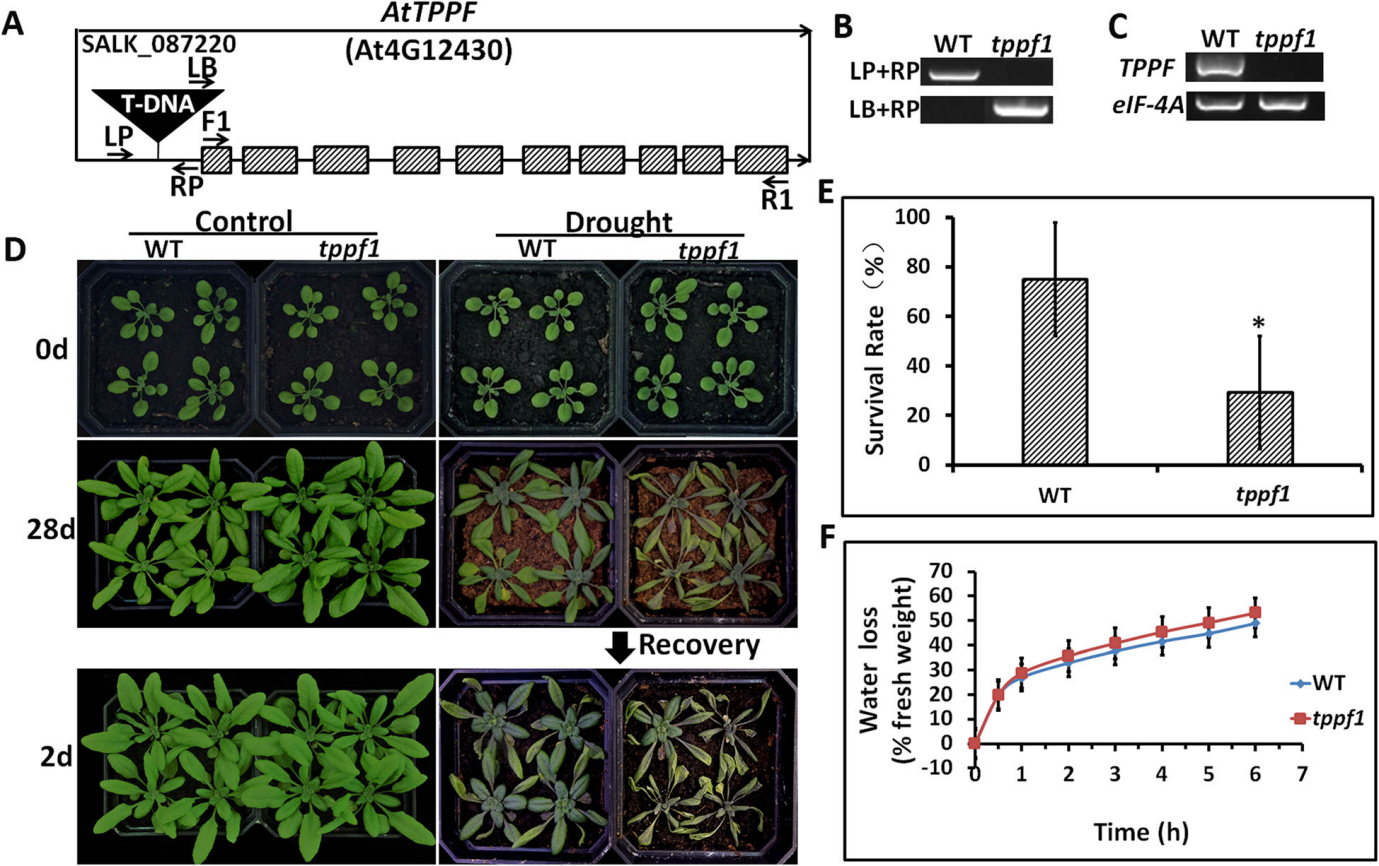
*tppf1* mutant is more sensitive to drought stress than wild-type (WT) *Arabidopsis thaliana.* **a** Structure of *AtTPPF* gene (*At4G12430*). Schematic illustrates T-DNA (black triangle) location within promoter and first intron (black line) in *AtTPPF* to form *tppf1* mutant. Arrows indicate positions of primers used in (**b**). **b** Homozygosity of T-DNA insertion in *tppf1* as assessed by PCR analysis of TPPF genomic fragment using left primer (LP) and right primer (RP), and for *tppf1* mutant using T-DNA insertion in conjunction with T-DNA-specific left border primer (LBb1.3) with RP. WT was Columbia-0. **c** *AtTPPF* transcript levels in WT and *tppf1* plants as determined by RT-PCR analysis with forward and reverse primers (TPPF1F and TPPF1R, respectively). *eIF-4A* served as reference standard. **d** Plant water-stress responses of 3-week-old WT and *tppf1* plants under long-day photoperiod (16-h light/8-h dark, 50% relative humidity). Six containers of each genotype (four plants per container) were evaluated in three independent experiments. Plants were exposed to drought stress by withholding water from d 0. Image shows results of one replicate from one experiment. **e** Plant survival rates at 4 d after re-watering. Plants were exposed to drought stress by withholding water from d 0, and remained unwatered until drought phenotype became visible. Plants were then re-watered and survival rate was determined. Six containers of each genotype (nine plants per container) were evaluated in five independent experiments. Error bars indicate SEs. Statistically significant differences were determined by Student’s t test (*p* < 0.05). **f** Water loss from detached leaves. Water losses from 0.5-g detached leaves from different plants were measured in triplicate at different times. Error bars indicate SEs. Statistically significant differences were determined by Student’s t test (*p* < 0.05)

A T-DNA insertional mutation in the promoter of *Arabidopsis AtTPPF* (*At4g12430*) in the Columbia-0 background was annotated as *tppf1* (SALK_087220) (Fig. 1a). We identified a homozygous *tppf1* line by PCR amplification using allele-specific primers (Fig. 1b; Additional file 5: Table S1). An RT-PCR analysis confirmed that there were *At4g12430* transcripts present in the WT but not in the *tppf1* mutant (Fig. 1c), suggesting that the mutation caused a loss of *AtTPPF* function. To investigate the genetic nature of *tppf1*, the mutant was back-crossed with WT Columbia-0. A phenotypic examination of the F_2_ progeny revealed a segregation ratio of 143 resistant: 45 sensitive plants (χ^2^ [3:1] = 0.258 < χ^2^_0.05_ = 3.84; *p* > 0.05), indicating that the *tppf1* mutation was inherited as a single recessive trait.

A phylogenetic analysis revealed that *AtTPPF* is highly similar to *OsTPP1* (Additional file 1: Figure S1), which increased drought tolerance when overexpressed in maize [20]. We then analyzed the drought tolerance of the *tppf1* mutant. Under a 28-d continuous drought treatment, the *tppf1* plants were more sensitive to drought stress than were WT plants, while the phenotypes of the *tppf1* plants were similar to those of WT plants under well-watered conditions (Fig. 1d). At 2 d after re-watering, the survival rate of the *tppf1* plants was significantly lower than that of WT plants (Fig. 1e). We evaluated the rate of water loss from detached leaves. Although the detached leaves of *tppf1* lost water slightly faster than did WT leaves, there was no statistical difference between their water loss rates (Fig. 1f).

### *AtTPPF* overexpression increases drought tolerance

**Fig. 2:**
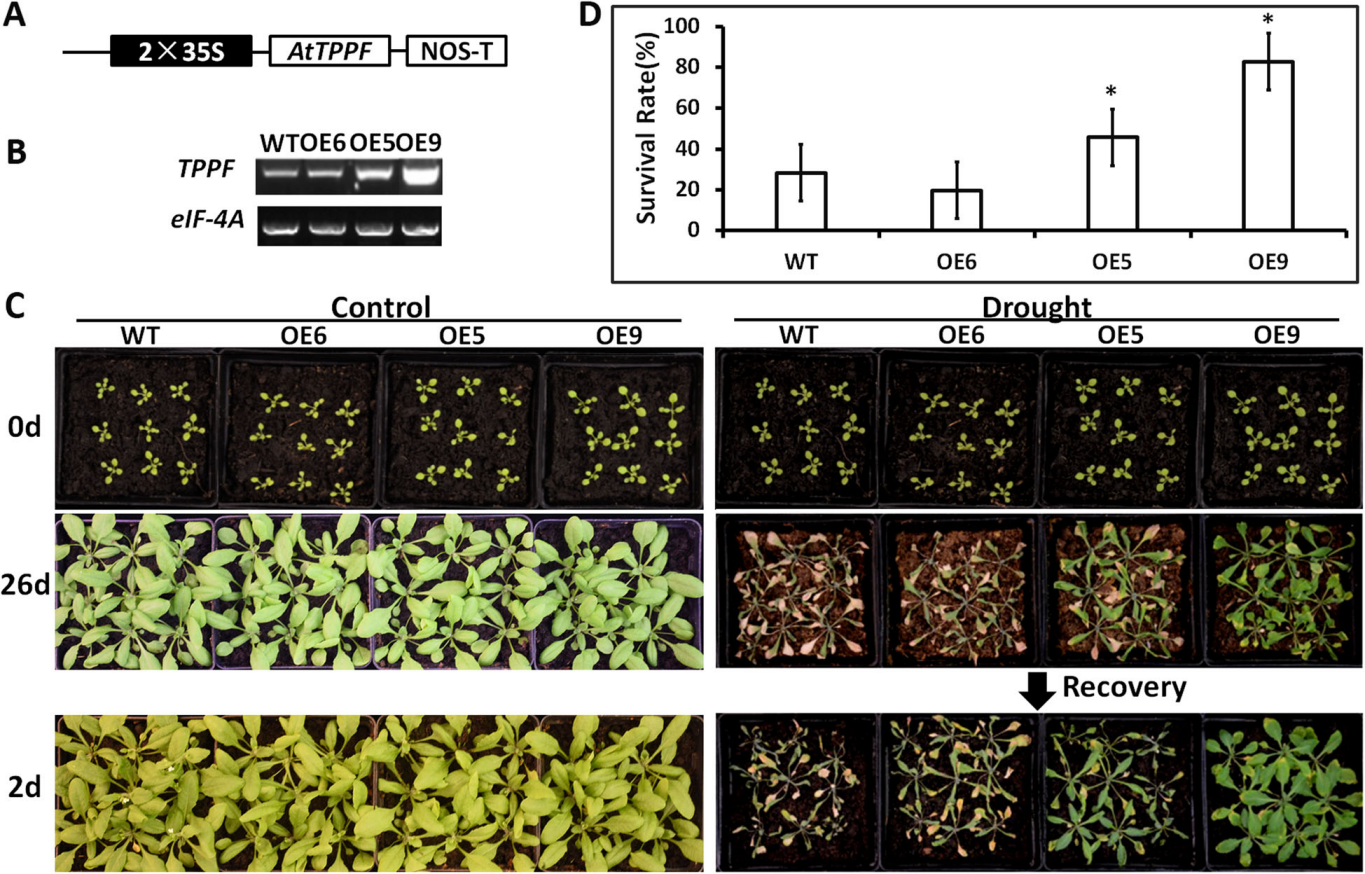
Overexpression of *AtTPPF* improves drought tolerance of *Arabidopsis thaliana.* **a** Schematic diagram of overexpression construct of *AtTPPF*. **b** RT-PCR analysis of *AtTPPF* gene transcripts in wild-type (WT) and three *AtTPPF*-overexpressing lines (OE6, OE5, and OE9). *eIF-4A* gene was used as internal control. **c** Analysis of drought-tolerance phenotypes of three overexpression lines in (**b**). Six containers of each genotype (nine plants per container) of 10-d-old plants growing in soil were subjected to drought by withholding water for 26 d. Three independent assays were performed, each producing similar results. **d** Plant survival rates at 2 d after rewatering. Plants in (**c**) were re-watered, and survival rates were determined by counting surviving plants after 2 d. Three independent experiments were performed, each containing approximately 81 plants. Data are means ± SEs. Different lowercase letters indicate significant differences among lines (Duncan’s test; *p* < 0.05)

To assess the function of *AtTPPF* under drought stress, we cloned the *AtTPPF* cDNA and generated an overexpression construct driven by the *CaMV 35S* promoter (Fig. 2a). This construct was then transformed into WT plants. Transgenic T3 homozygous lines in which *AtTPPF* expression levels were greater than that of WT plants were obtained. Three lines with different expression levels (OE6, OE5, and OE9) were selected for further analyses (Fig. 2b). The three lines displayed varying degrees of drought resistance, proportional to their transcript levels of *AtTPPF* (Fig. 2c). For example, OE9 plants showed the highest *AtTPPF* expression levels, and grew vigorously after 26 d of drought. The *AtTPPF* expression levels were relatively lower in OE5 plants, which grew less vigorously than the OE9 plants. The WT and OE6 plants displayed severe wilting symptoms under drought stress (Fig. 2c). The OE5 and OE9 plants had significantly greater survival rates (45.7 and 82.7%, respectively) than that of WT plants (28.4%) under drought stress (Fig. 2d). Thus, overexpression of *AtTPPF* increased the drought tolerance of plants, and the level of improvement was proportional to the level of *AtTPPF* gene expression. Because the overexpression of *AtTPPF* increased drought tolerance and the loss of function of *AtTPPF* compromised drought-stress tolerance, we concluded that *AtTPPF* positively regulates drought tolerance in *Arabidopsis*.

To confirm the function of *AtTPPF* in the drought response, we performed a complementation experiment with the *tppf1* mutant using the same construct as described above. The *CaMV 35S* overexpression construct was transformed into the *tppf1* mutant. Eight T2 transgenic lines were obtained, and complemented lines with *AtTPPF* expression levels similar to that of WT were selected for further analyses. The phenotypes of the complemented lines were similar to those of WT plants under drought conditions (Additional file 2: Figure S2). These results confirmed that the loss-of-function mutation of *AtTPPF* conferred drought sensitivity in *Arabidopsis.*

### DREB1A binds to drought-responsive DRE/CRT element in *TPPF* promoter

**Fig. 3:**
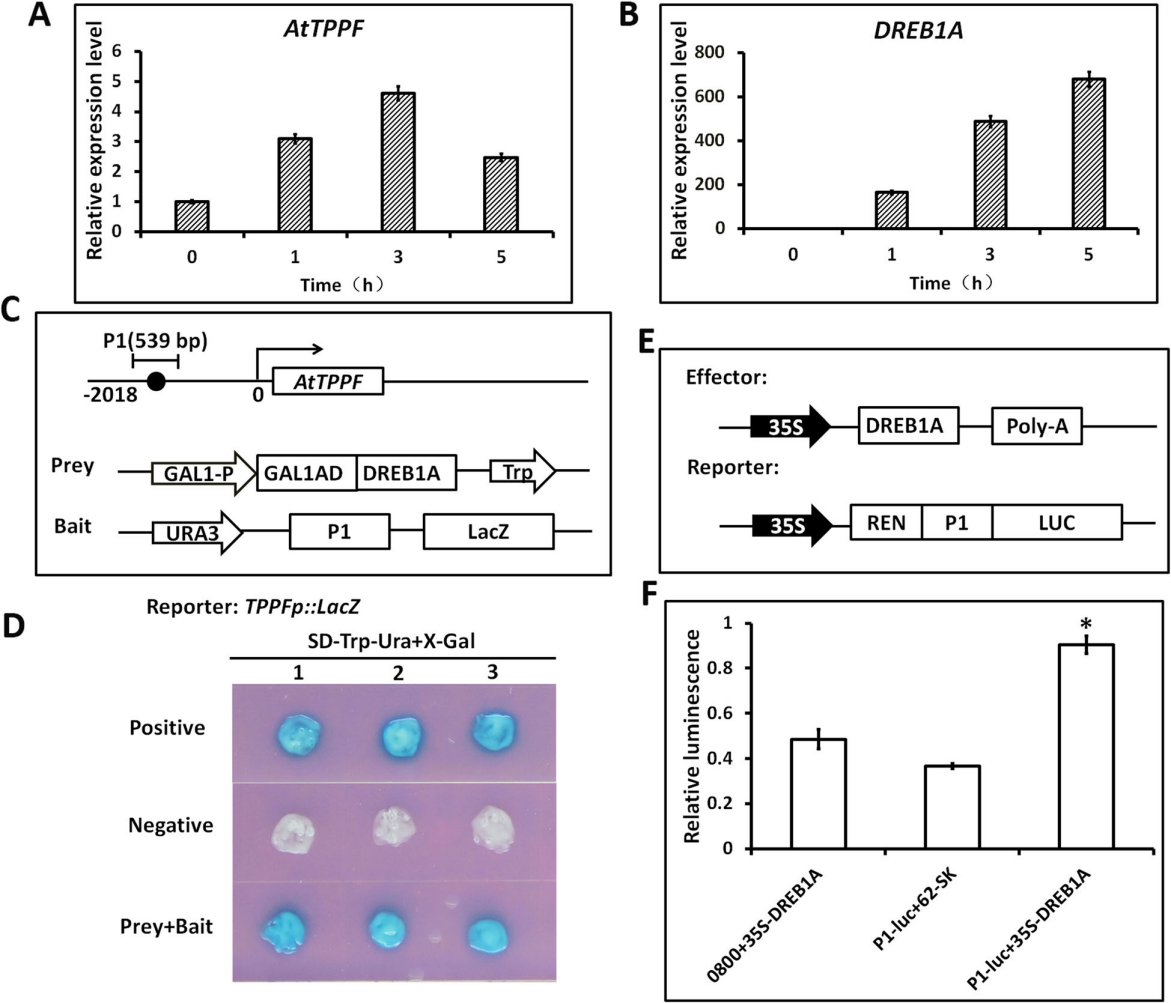
DREB1A binds to *AtTPPF* promoter and acts as a transcriptional activator. **a** Transcript levels of *AtTPPF* in wild-type (WT) plants under drought conditions. **b** Transcript levels of *DREB1A* in WT plants under drought conditions. **c** Schematic diagrams of *AtTPPF* promoter and effector and reporter constructs used for yeast one-hybrid (Y1H) assays. Black circle indicates DRE/CRT “ACCGAC” *cis*-acting element in promoter. P1 indicates partial promoter fragment used to construct bait plasmid. **d** Results of Y1H assays and growth of yeast cells. Positive control: *AtTPPF-P*, full-length promoter (P) of *AtTPPF*, cotransformed with *pJG4–5* empty vector; negative control: bait/*pJG4–5*, prey+bait, cotransformed with prey and bait on selective medium supplemented with X-Gal. **e** Schematic diagrams of effector and reporter constructs used in transactivational analyses. Promoter fragment P1 was inserted into reporter vector *pGreen II 0800-LUC*, and Renilla luciferase (REN) was used as control for activity normalization. DREB1A was inserted into *pGreen II 62-SK* expression vector. **f** Transient expression analysis of DREB1A. Transient expression assay in tobacco (*Nicotiana benthamiana*) to examine interaction between DREB1A and *AtTPPF* promoter. Promoter activities, (LUC:REN ratio) in tobacco leaf discs cotransformed with effector and reporter constructs. Control, LUC:REN ratio in leaf discs transformed with an empty vector (*pGreen II 62-SK*/*pGreen II 0800-LUC*). Statistically significant differences were determined by Student’s t test (*p* < 0.05)

Searches of the promoter region of *AtTPPF* (the 2-kb region upstream from the transcriptional initiation site) revealed a DRE/CRT cis-acting element, “ACCGAC,” and three cis-acting ABA-responsive elements (ABREs). Because DRE/CRT elements can interact specifically with DREB/CBF transcription factors, we analyzed coexpression models of *AtTPPF* and the DREB-coding genes *DREB1A*, *DREB2A,* and *DREB1B*, using the AtGenExpress Visualization Tool. The expression of *AtTPPF* was essentially consistent with that of *DREB1A* (Additional file 3: Figure S3). To confirm this coexpression pattern, we used RT-qPCR to quantify the transcript levels of *AtTPPF* and *DREB1A* under drought conditions. Both genes were induced by drought, and their expression patterns were essentially similar (Fig. 3a and b). These data suggest that DREB1A may activate the expression of *AtTPPF* by binding to the DRE/CRT motif.

To determine whether DREB1A recognizes the *AtTPPF* promoter in vivo, we performed a yeast one-hybrid assay to determine whether DREB1A can directly bind to the DRE/CRT “ACCGAC” motif in the *AtTPPF* promoter region. The full-length 2-kb promoter of *AtTPPF* (including DRE/CRT and three ABRE motifs) was cloned into a bait construct. Interestingly, a blue colony without any prey proteins was identified, indicating that the full-length fragment was autonomously activated (Additional file 4: Figure S4). When a 539-bp P1 fragment containing the DRE/CRT motif and the ABRE motif was cloned into the bait construct (Fig. 3c), blue colonies were observed only in the presence of DREB1A prey (Fig. 3d), indicating that DREB1A could recognize and bind to the “ACCGAC” motif in the *AtTPPF* promoter. However, when AREB1 (binding to the ABRE motif) was added as prey, no blue colonies were obtained (Additional file 4: Figure S4), indicating that there was no interaction between the P1-containing ABRE motif and AREB1. These results indicate that the *AtTPPF* promoter is recognized specifically by DREB1A.

*DREB1A* encodes a member of the DREB subfamily of the ERF/AP2 family of transcriptional activators [23]. To determine whether DREB1A can activate the expression of *AtTPPF*, we performed a dual luciferase (LUC) assay to analyze reporter gene expression. The P1 fragment was introduced into the *pGreen II 0800-LUC* vector to generate a reporter construct, and the cDNA of *DREB1A* was inserted into *62-SK* to generate an effector construct. Tobacco (*Nicotiana benthamiana*) leaves were cotransformed with both the effector and reporter constructs and then relative LUC activity was determined. As expected, LUC activity was higher in the presence of both the effector and reporter constructs, including both 0800 + *35S*-*DREB1A* and *P1:luc* + *62-SK* (Fig. 3d, e), than in the presence of the negative control. This result implies that DREB1A can act as a transcriptional activator of *AtTPPF*.

### DREB1A positively modulates *AtTPPF* expression

**Fig. 4:**
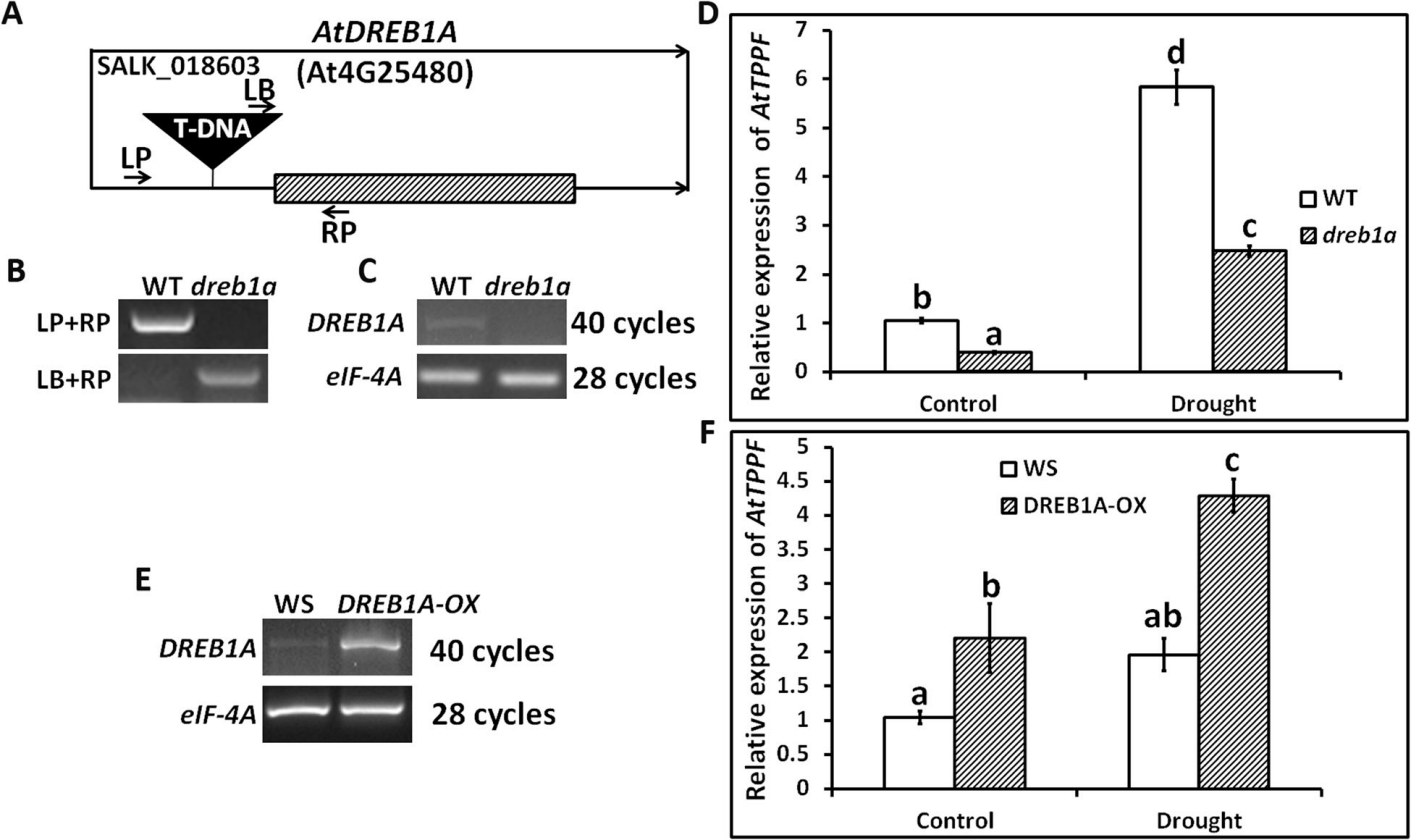
*DREB1A* modulates expression of *AtTPPF* in vivo*.* **a** Schematic illustrating T-DNA location in 5′- untranslated region of *AtDREB1A* to form *dreb1a*. Arrows indicate positions of primers used in (**b**) and (**c**). **b** Homozygosity of T-DNA insertion in *dreb1a* as assessed by PCR analysis of DREB1A genomic fragment using left primer (LP), right primer (RP), and T-DNA-specific left border primer (LB). **c** *DREB1A* transcript levels in wild-type (WT) and *dreb1a* plants as determined by RT-PCR analysis with forward and reverse primers (LP and RP, respectively). *eIF-4A* served as reference standard. **d** *DREB1A* transcript levels in WS and *DREB1A-*OE (*DREB1A-OX*) plants as determined by RT-PCR analysis with forward and reverse primers (LP and RP, respectively). *eIF-4A* served as reference standard. **e** Relative abundance of *AtTPPF* transcripts in WT and *dreb1a* plants under control and drought conditions. Three independent experiments were performed. Error bars indicate SEs (*n* = 4). Different lowercase letters (a, b or c, d) show significant differences among lines (Duncan’s test; *p* < 0.05). **f** Relative abundance of *AtTPPF* transcripts in WS and *DREB1A-OX* plants under control and drought conditions. Three independent experiments were performed. Error bars indicate SEs (*n* = 4). Different lowercase letters (a and b or c and d) indicate significant differences among lines (Duncan’s test; *p* < 0.05)

To further confirm the direct regulation of *AtTPPF* by DREB1A, we acquired related *DREB1A* lines. These lines included one T-DNA insertional mutant (*dreb1a*) in the untranslated region of *DREB1A* (*At4g25480*) in the WT Columbia-0 background (SALK_018603) (Fig. 4a); and one overexpression line of *DREB1A* in the Wassilewskija (WS) background (CS69502). The homozygous *dreb1a* line was identified by PCR amplification using allele-specific primers (Fig. 4b). An RT-PCR analysis confirmed that no *DREB1A* transcripts were present in the *dreb1a* mutant compared with WT (Fig. 4c). Compared with WS plants, DREB1A-overexpression plants (DREB1A-OX) showed higher transcript levels of *DREB1A* (Fig. 4d).

Previously, we confirmed that DREB1A could activate *AtTPPF* transcription (Fig. 3). Consequently, we performed expression assays of *AtTPPF* in both *dreb1a* mutants and DREB1A-OX plants. As expected, the transcript abundance of *AtTPPF* was much lower in the *dreb1a* plants than in WT plants under both well-watered control and drought conditions. Thus, the loss of *DREB1A* led to reduced *AtTPPF* expression. In contrast, compared with WS plants, the DREB1A-OX plants showed increased transcript levels of *AtTPPF* under both control and drought conditions. Thus, we concluded that DREB1A plays a positive role in *AtTPPF* expression.

### *AtTPPF* regulates trehalose and H_2_O_2_ levels in response to drought stress

**Fig. 5:**
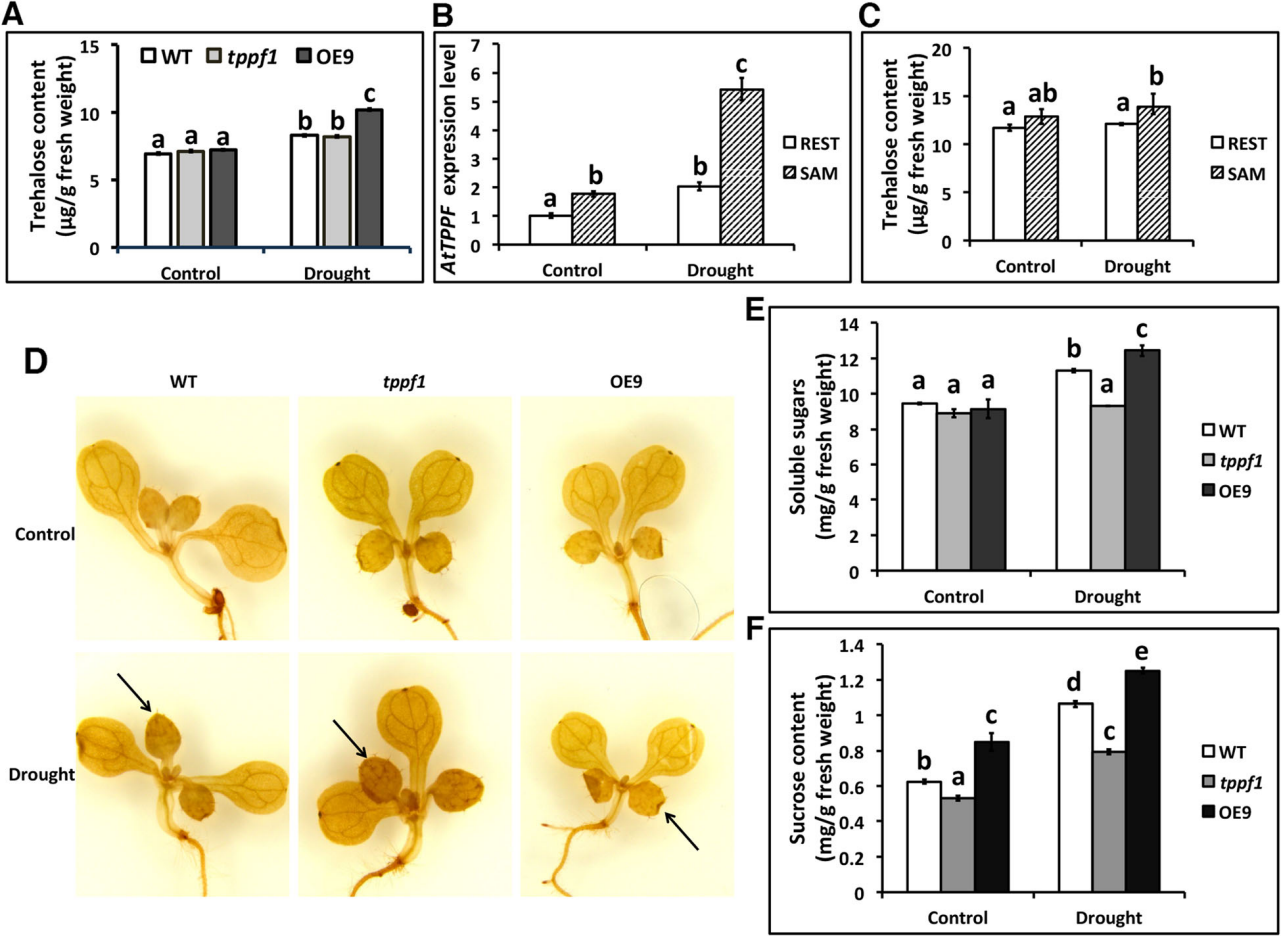
Trehalose, ROS (H_2_O_2_), and sucrose contents in wild-type (WT) and mutant/OE lines. **a** Trehalose contents in the whole seedlings of WT, *tppf1* mutant, and OE9 lines under control and drought-stress conditions. Error bars indicate SEs (*n* = 9). Different lowercase letters (a and b or c and d) indicate significant differences among lines (Duncan’s test; *p* < 0.05). **b** Relative abundance of *AtTPPF* transcripts in REST (the rest part of the whole seedlings apart from the SAM) and SAM (shoot apical meristem) of WT plants under control and drought conditions. Error bars indicate SEs (*n* = 4). Different lowercase letters (a and b or c and d) indicate significant differences among lines (Duncan’s test; *p* < 0.05). **c** Trehalose contents in SAM and REST under control and drought conditions. Error bars indicate SEs (*n* = 3). Different lowercase letters (a and b or c and d) indicate significant differences among lines (Duncan’s test; *p* < 0.05). **d** H_2_O_2_ contents in 10-d-old seedlings on MS treated without (Control) or with PEG6000 (Drought) for 24 h and subjected to DAB staining. Three biological repeats were analyzed. Black arrows indicate new leaves. **e** Soluble sugars contents in 10-d-old WT, *tppf1* mutant, and OE9 plants with or without drought treatment. Error bars show SEs (*n* = 4). Different lowercase letters (a, b, or c) indicate significant differences among lines (Duncan’s test, *p* < 0.05). **f** Sucrose contents in 10-d-old WT, *tppf1* mutant, and OE9 plants with or without drought treatment. Error bars indicate SEs (*n* = 4). Different lowercase letters (a, b, c, d or e) indicate significant differences among lines (Duncan’s test; *p* < 0.05)

*AtTPPF* encodes a protein in the haloacid dehalogenase-like hydrolase superfamily that is involved in trehalose biosynthesis. Thus, we monitored changes in the trehalose content before and after the drought treatment. The trehalose contents did not increase in the WT and *tppf1* mutants, and but accumulated to higher levels in OE9 plants than in WT or *tppf1* mutants under drought conditions (Fig. 5a). These results indicate that *AtTPPF* overexpression leads to the accumulation of trehalose in response to drought stress.

*AtTPPF* is expressed in the shoot apical meristems (SAM) [16], so we measured the transcript levels of *AtTPPF* in the SAM and the REST (the rest part of whole seedling apart from the SAM). As expected, there was a significant difference in *AtTPPF* transcript abundance between the SAM and the REST under both control and drought conditions (Fig. 5b), indicating that *AtTPPF* is preferentially expressed in the SAM. The distinct difference in transcript abundance levels occurred specifically under drought conditions, suggesting that *AtTPPF* plays a key role in the response to drought stress in the SAM. We did not find an appreciable increase in the trehalose levels in whole seedlings of WT and *tppf1* under drought stress. Therefore, we measured the trehalose content in the SAM to detect subtle changes. We measured the trehalose contents in the SAM and the REST under control and drought conditions, and found limited differences in trehalose levels in the SAM between the two conditions (Fig. 5c). Although trehalose accumulated in the OE9 plants under drought conditions (Fig. 5a), the increase was relatively small. Thus, this may not be the only explanation for the elevated drought tolerance of *AtTPPF*-overexpressing plants. Low trehalose levels are not sufficient to control osmoregulation [3, 24]. Therefore, other factors may be responsible for the improved drought tolerance conferred by *AtTPPF* overexpression.

In plants, H_2_O_2_ is a major reactive oxygen species (ROS) that functions as an important messenger to relay drought stress signals [25]. Because *AtTPPF* was expressed at relatively high levels in the SAM of young seedlings (Fig. 5b), we measured H_2_O_2_ levels using DAB staining to verify the possible functions of *AtTPPF* in scavenging ROS in the SAM. Compared with WT plants, the OE9 plants accumulated less H_2_O_2_, especially in new leaves, after 24 h of drought treatment. In contrast, the *tppf1* mutants accumulated more H_2_O_2_ under the same conditions (Fig. 5d). Even though there was no difference in trehalose levels between the *tppf1* mutant and WT plants, the loss of function of *AtTPPF* caused a partial failure of ROS scavenging. These results indicate that *AtTPPF* may play roles in antioxidant systems to protect plants under drought conditions. Soluble sugars are known to be involved in redox-balancing in plants [11], so we determined the total soluble sugars contents. As shown in Fig. 5e, compared with the WT, OE9 plants accumulated more soluble sugars, while *tppf1* mutants accumulated less soluble sugars, under drought conditions. In plants, T6P plays a significant role in regulating metabolism and development [5, 8]. The overexpression of *TPP1* in maize is associated with changes in T6P and sucrose levels [20]. As shown in Fig. 5f, sucrose accumulated to higher levels in OE9 plants than WT plants under control and drought conditions, but did not accumulate in *tppf1* mutants. Therefore, *AtTPPF* expression could lead to increased sucrose levels.

### Transcriptome analysis of WT and *AtTPPF* OE plants under drought conditions

**Fig. 6:**
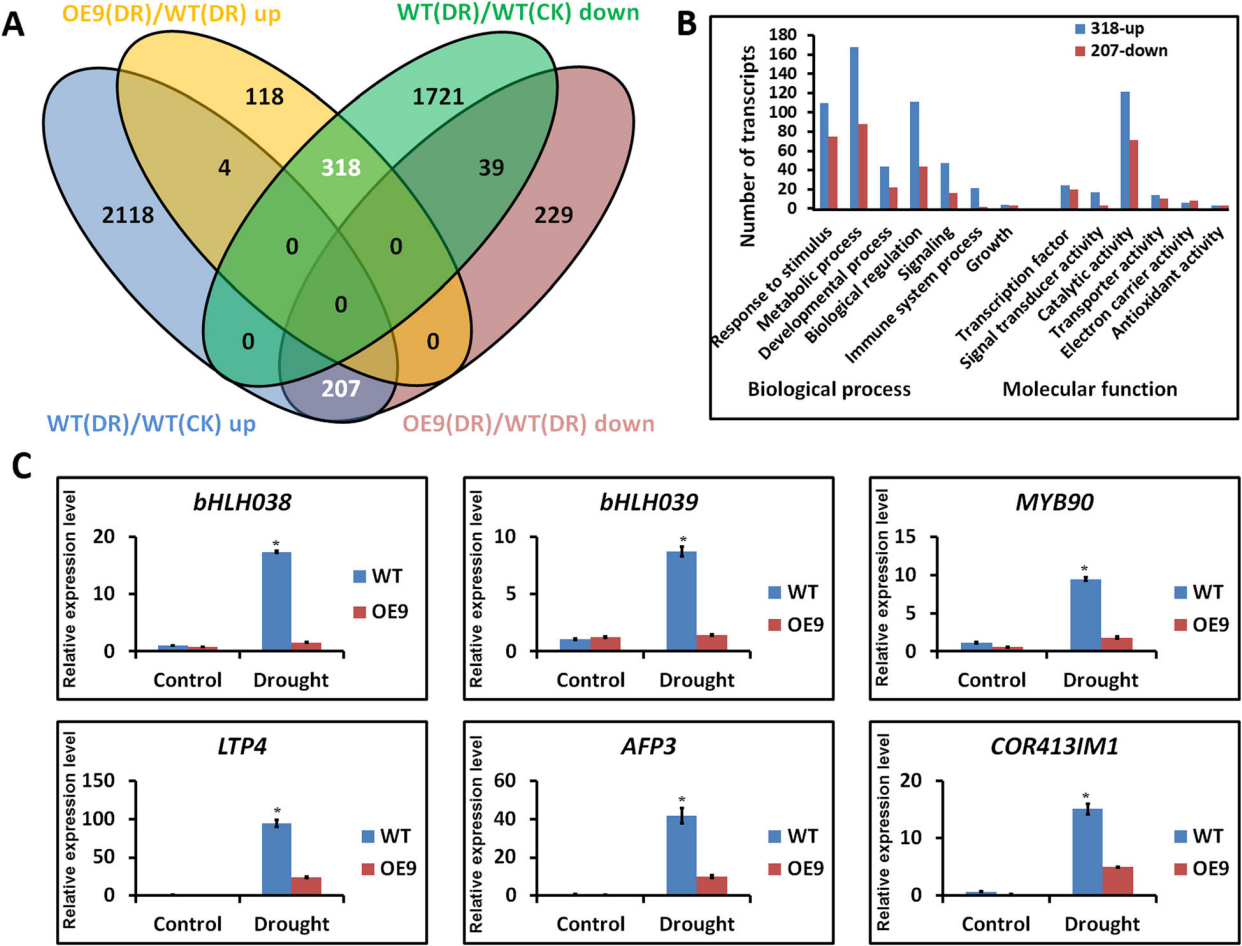
Changes in gene expression levels in *tppf1* as assessed by RNA-seq. **a** Venn diagram showing overlapping differentially expressed gene (DEG) sets among OE9(DR)/WT(DR) up (OE9-drought vs. WT-drought; upregulated), OE9(DR)/WT(DR) down (OE9-drought vs. WT-drought; downregulated), WT(DR)/WT(CK) up (WT-drought vs. WT-well-watered; upregulated), and WT(DR)/WT(CK) down (WT-drought vs. WT-well-watered; downregulated). **b** Statistical analysis of upregulated and downregulated genes between OE9 and WT plants under well-watered [CK/CK (OE9/WT)] and drought conditions [DR/DR (OE9/WT)]. **c** Results of RT-qPCR analysis of related genes. Error bars indicate SEs (*n* = 4, four biological replicates). All values for WT and OE9 plants are statistically significantly different from each other (Student’s t test; *p* value < 0.05)

To further evaluate the role of *AtTPPF* in modulating plant responses to drought stress on a broader scale, we analyzed the transcriptomes of WT and OE9 plants. Total RNAs from 2-week-old seedlings under well-watered and drought conditions were isolated and subjected to RNA-seq, with three biological replicates. A total of 3 GB of clean data was obtained, which was mapped to the gene models in the TAIR 10 assembly [26]. The fragments per kilobase of transcript per million mapped reads (FPKM) values of each gene were calculated for the WT and OE9 plants. By using pairwise comparisons between the WT and OE9 plants, we defined differentially expressed genes (DEGs) as those whose FPKM values changed by at least two-fold (*p* value < 0.05). A comparative analysis was performed to compare the WT and OE9 plants under well-watered and drought conditions. Among the DEGs (OE9 vs. WT), 440 were upregulated under drought conditions and 475 were downregulated. We expected that some of these genes would contribute to the improved drought tolerance of the *AtTPPF*-overexpressing plants (Fig. 6a). Interestingly, 318 (72%) of the 440 upregulated genes in OE9 were repressed in WT plants under drought conditions (Fig. 6a), while 207 (44%) of the 475 downregulated genes in OE9 were induced in WT plants under drought conditions (Fig. 6a). A possible explanation for these results is that the elevated trehalose levels in the SAM may relieve severe effects of drought by modulating the expression of drought-responsive genes.

To test this idea, we performed a gene functional annotation of the 318 upregulated (ERG-U) and 207 downregulated (DIG-D) genes in the OE9 plants (Additional file 7: Table S3 and Additional file 8: Table S4). A gene ontology (GO) analysis revealed that the 318 and 207 genes were involved in various biological and molecular processes (Fig. 6b), especially in responses to stimuli and metabolic processes. The functional annotation analyses suggested that a large portion of these genes encoded transcription factors and enzymes (Fig. 6b). Drought induces plant stomatal closure and reduces photosynthesis [27]. Among the 318 ERG-Us, three were associated with electron transport activity: *ENH1*, encoding a rubredoxin family protein involved in metal ion binding; *CYP81D1*, encoding a member of the *CYP81D* family of cytochrome p450s; and *CYP71B13*, encoding a putative cytochrome P450. Some genes were involved in carbohydrate metabolism, such as *SFP2*, which encodes a sugar-porter family protein that participates in sugar transport, as well as *SUC1* and *SUC5*, encoding members of the sucrose-proton symporter family that are involved in sucrose metabolism and transport. The increased expression levels of these genes in OE9 plants may enhance photosynthesis and maintain the carbohydrate balance under drought conditions. In addition, a cell wall biogenesis gene, *XTR8*, was identified. The product of this gene is involved in cell wall modification and improves cell wall stiffness, further affecting changes in ion leakage [28]. The ERG-Us also included *FSD3*, encoding a type of peroxidase; FSD3 scavenges ROS and may improve the ability of OE9 plants to remove ROS under drought conditions [29]. This hypothesis was supported by the patterns of ROS accumulation in the various lines (Fig. 5d). Thus, the increased drought tolerance of the OE9 plants was related to increased transcript levels of genes that are normally drought-repressed genes in WT plants under drought stress.

Surprisingly, the 207 DIG-Ds included many stress-responsive transcription factors, including MYB, WRKY and basis helix–loop–helix (bHLH) types. Specifically, these included *MYB90*, which regulates anthocyanin synthase, and affects anthocyanin accumulation under adverse conditions [30]; and *bHLH38* and *bHLH39,* which are involved in improving iron homeostasis [31]. The DIG-Ds also included drought-related genes, such as *LTP3*, *LTP4,* and *COR413IM1*; and the ABA signaling-regulated gene *AFP3*. Because the transcript levels of genes that are drought-induced in WT were downregulated in the OE9 plants, we hypothesized that the overexpression of *AtTPPF* contributed to drought responses by stimulating vigorous growth during drought. To verify the RNA-seq data, we performed quantitative RT-PCR to measure the expression levels of several of the annotated genes (Fig. 6c). The results were consistent with those obtained from the RNA-seq data.

## Discussion

### *AtTPPF* increases drought tolerance through accumulation of soluble sugars

Previous studies on the *TPP* family have focused mainly on their functions in *Arabidopsis* growth and development and in improving crop production [2, 16, 21]. They have been the subject of practical field trials in certain crop species, including wheat and rice [19, 20]. For example, the *OsTPP1* gene was shown to increase drought tolerance and yields when overexpressed in maize [20]. As a member of the *TPP* family in *Arabidopsis*, the *AtTPPF* gene is highly homologous to *OsTPP1*, which is induced specifically under dehydration conditions [22]. In this study, the expression of *AtTPPF* was induced by drought (Fig. 3a). The loss of function of *AtTPPF* led to drought sensitivity, while its overexpression increased drought tolerance (Figs. 1 and 2). These results indicate that *AtTPPF* plays a positive role in drought tolerance.

Trehalose is a non-reducing disaccharide that functions as an osmoprotectant in the maintenance of cellular osmotic balance [32]. Trehalose is thought to act as an osmolyte that protects resurrection plants, such as *Selaginella* spp. [33] and *Myrothamnus flabellifolius* [34], from desiccation. Under dehydration conditions, trehalose plays a role in stabilizing dehydration-related enzymes and proteins, as well as lipid membranes, and it can scavenge ROS to protect biological structures from damage [35]. As a non-reducing disaccharide, trehalose is stable and unreactive. It accumulates to high levels in fungi, bacteria, insects, and arthropods, in which it plays a dual role as a carbon storage molecule and a protective compound in certain situations. Trehalose may also be a target to improve drought tolerance in plants. Increasing the expression of *TPP* in *Arabidopsis* led to a four-fold increase in trehalose contents [1, 36]. Our results showed that the trehalose content is very low in *Arabidopsis* (less than 10 μg/g FW, even under drought conditions), consistent with a previous report [24]. The loss of function of *AtTPPF* did not affect the trehalose content under either well-watered or drought conditions, consistent with a previous study [16]. This may result from the complementary effects of other genes, such as *AtTPPG* (the duplicate of *AtTPPF*). However, the overexpression of *AtTPPF* resulted in a 25% increase in the trehalose content under drought conditions, compared with the level in WT plants (Fig. 5a). Even though we detected a slight effect of *AtTPPF* as an osmotic protector, the OE9 plants showed increased ROS scavenging abilities (Fig. 5d). The enhanced soluble sugars level under drought conditions may also contribute to osmotic protection (Fig. 5e). Thus, the overexpression of *AtTPPF* increased the levels of soluble sugars, which could subsequently protect the cell from oxidative damage and enhance drought tolerance.

### *AtTPPF*-overexpressing plants maintain carbohydrate metabolism and exhibit decreased drought sensitivity under drought stress

We detected no correlation between the prominent drought-tolerant phenotype and limited trehalose accumulation, suggesting that some other pathway might be responsible for the increased drought tolerance conferred by *AtTPPF*. Therefore, we performed a comparative transcriptional analysis of OE9 and WT plants under well-watered and drought conditions. The overexpression of *AtTPPF* altered the transcript levels of numerous genes encoding transcription factors and enzymes under drought conditions (Fig. 6b). Among the ERG-Us, 318 were upregulated in the OE9 plants during drought stress. They encoded proteins involved in electron transport activity, carbohydrate metabolism, cell wall modification, and ROS scavenging. Thus, the overexpression of *AtTPPF* may contribute to increasing photosynthesis, protecting biological components from damage caused by ROS, and maintaining carbohydrate metabolism under drought conditions. The 207 genes that were downregulated in the OE9 plants under drought stress included numerous stress-related transcription factor genes and drought-related genes. This result indicates that the increased expression of *AtTPPF* reduces the negative effects of drought stress by some unknown mechanism. Considering the relatively low accumulation of H_2_O_2_ and the relatively low expression levels of drought-induced regulators in OE9 plants under drought, this relief of the negative effects of drought may partly result from ROS removal, which would improve the osmotic regulation of OE9 plant cells. Thus, increasing the expression of *AtTPPF* could maintain the balance between gene expression and metabolism, as well as increasing adaptability to drought conditions.

### *AtTPPF* is a direct downstream target of DREB1A

Although the overexpression of TPS and/or TPP has been widely used to increase plant drought tolerance [6, 19, 20, 36], the underlying molecular mechanisms involved in the dynamic regulation of genes in the trehalose biosynthetic pathway under drought-stress conditions are largely unknown. DREB1A is a DREB/CBF transcription factor that interacts specifically with the DRE/CRT *cis*-acting element and regulates the expression of many stress-inducible genes to increase the stress tolerance of *Arabidopsis* [37]. In *Arabidopsis,* DREB1A expression can improve abiotic stress tolerance by increasing late embryogenesis-abundant protein levels and compatible solute contents [22, 38]. In another study, trehalose levels were significantly increased in WT and *35S:DREB1A* transgenic plants grown under drought conditions for 2–3 d [22]. Here, the expression of *AtTPPF* during drought stress was consistent with that of *DREB1A* (Fig. 3a, b). Yeast one-hybrid assays revealed that DREB1A could bind directly to the drought-responsive DRE/CRT element in the *AtTPPF* promoter, and LUC activity assays further indicated that DREB1A could positively activate *AtTPPF* gene expression. Furthermore, the transcript abundance of *AtTPPF* was significantly lower in the *dreb1a* mutant and much higher in the DREB1A-OX plants than in WT (Fig. 4e, f). Although the expression of *AtTPPF* was also induced in the *dreb1a* mutant under drought conditions, this phenomenon may result from the existence of other modulatory genes. Thus, *AtTPPF* appears to be a direct downstream target of DREB1A, and *AtTPPF* expression is positively modulated by DREB1A in plants.

**Fig. 7:**
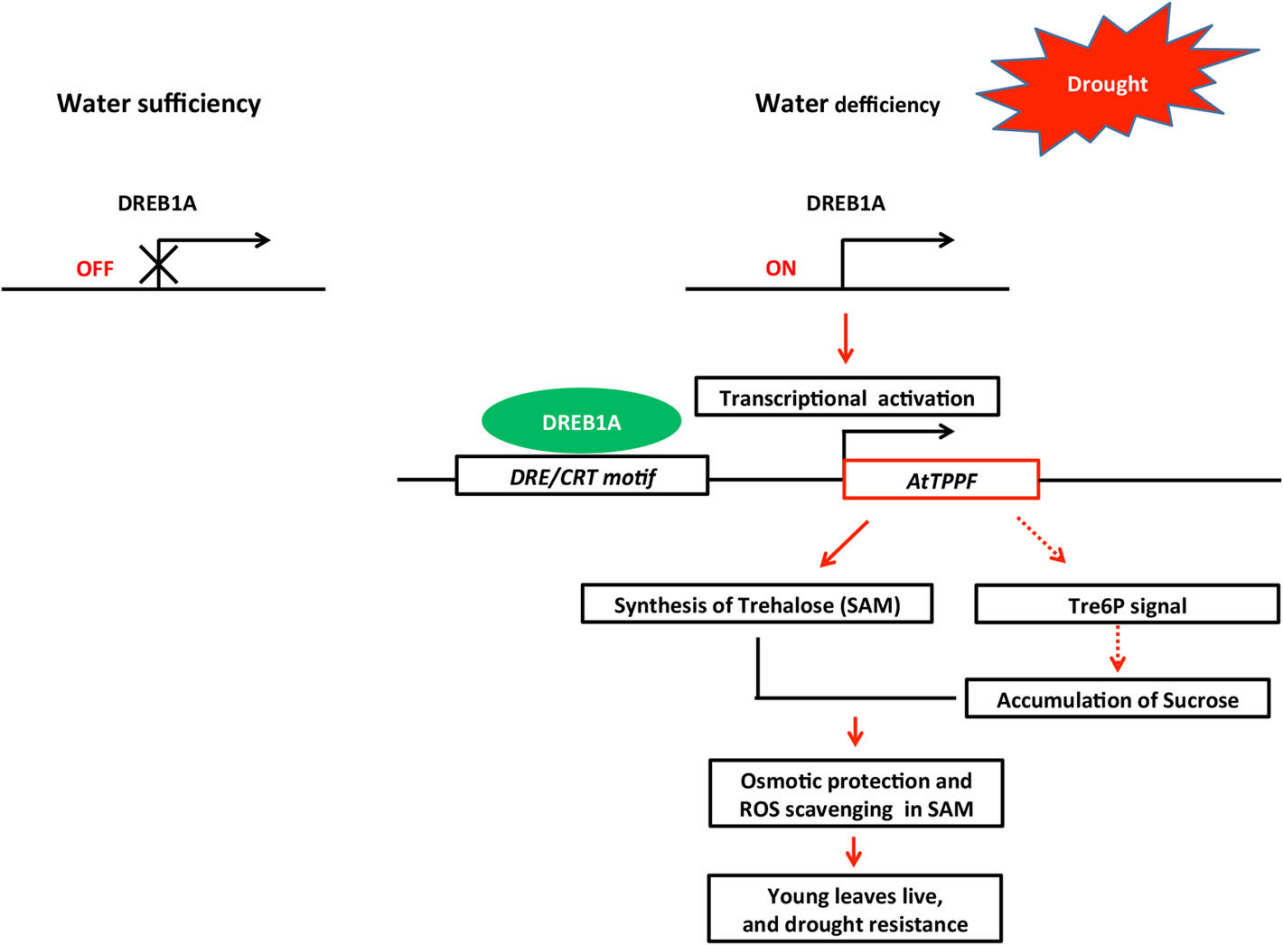
Proposed model of DREB1A-regulated *AtTPPF* drought-response network. Under well-watered conditions, DREB1A is in “OFF” position. Under drought stress, expression of DREB1A is induced to bind to DRE/CRT motif in promoter of *AtTPPF,* thereby activating *AtTPPF* transcription. Increased expression of TPPF most likely affects T6P signal, which may be a causal factor in the increase of soluble sugars, which function as osmotic protectors and scavengers of reactive oxygen species in the shoot apical meristem. This helps young leaves survive under long-term drought conditions

We developed a hypothetical response model for *AtTPPF* during drought stress (Fig. 7). Under normal conditions, there is sufficient water to meet all the needs of the plant, and the stress-induced gene *DREB1A* is in the “OFF” position. When the plant encounters drought stress, *DREB1A* switches to the “ON” position. DREB1A is expressed at high levels, and binds to the DRE/CRT motif in the promoter of *AtTPPF* and activates its transcription. The increased expression of *AtTPPF* most likely affects the T6P signal, which may lead to an increase in soluble sugars. Soluble sugars function as osmolytes to protect the cells of young leaves from ROS damage and allow them to complete their life cycle normally (Fig. 7).

## Conclusions

Our results show that *AtTPPF* is related to changes in soluble sugar levels under drought conditions. *AtTPPF*-overexpressing plants show increased levels of soluble sugars, which may function as osmolytes to protect cells from ROS damage. Our results also show that DREB1A can directly interact with the *AtTPPF* promoter and activate *AtTPPF* transcription under drought conditions. These findings shed light on the transcriptional regulation of *AtTPPF* and also provide a practical tool for improving the drought tolerance of plants through the manipulation of *AtTPPF* expression.

## Methods

### Plant materials and growth conditions

The ecotype used in the article is Col-0. T-DNA insertion loss-of-function *tppf1* mutant seeds (SALK_087220) were kindly supplied by Professor Yuan Qin from Center for Genomics and Biotechnology, Haixia Institute of Science and Technology of Fujian Agriculture and Forestry University. Seeds of the *dreb1a* T-DNA insertion line (SALK_018603) and the DREB1A-OX line (CS69502) were ordered from the Arabidopsis Biological Resource Center (ABRC). Single-copy and homozygous T3 plants were identified by genetic segregation on agar medium that contained kanamycin. Stratified seeds were transferred either to Murashige and Skoog (MS) medium or to the soil and cultivated at 22 °C under a 16-h light/8-h dark photoperiod at 80 μmol quanta m^− 2^ s^− 1^ light intensity and 50% relative humidity.

For *tppf1* T-DNA insertion identification, total genomic DNA was extracted from the leaves, and PCR was performed using primer pairs: LP: 5′-TTCACCTGCACACAACAAATC-3′, RP: 5′-CCCAACATGGATGGTTCTTC-3′ and LB: 5′-ATTTTGCCGATTTCGGAAC-3′.

For the identification of *dreb1a* T-DNA insertion identification, the PCR primer pairs used are: LP: 5′-GCCACACATTCATACGCAAAGA-3′, RP:5′- AGCTCGAGCTGCCATCTCAGCG-3′and LB: 5′-GCGTGGACCGCTTGCTGCAACT-3′.

### Drought stress analysis

One-week-old wild-type (Col-0), *35S:AtTPPF* transgenic, *tppf1* mutant plants and the complementary plants grown in soil (PINDSTRUP from Denmark, pH 5.0, 0–10 mm, using the same weight of black plastic pot 10 cm × 10 cm, dry weight of soil: 100 g ± 0.1 g, and make sure the filled water is the same) in a growth room (16 h light/8 h dark, 22 °C, 50% humidity) were subjected to drought stress by withholding water for approximately 26 d (the duration ranged from 14 to 26 d according to the growth conditions and the number of seedlings per container in each experiment, for the *tppf1* mutant drought assay, 4 seedlings planted in a container, the drought days are 28 d; for the *35S:AtTPPF* transgenic lines drought assay, 9 seedlings planted in a container, the drought days are 26 d; and for the complementary experiments, 5 seedlings planted in a container, the drought days are 14 d). Once differences were apparent in each experiment, the plants were re-watered, after which their survival rates were determined after 2 d.

### Plasmid constructs and plant transformation

Total RNA was extracted from 2-week-old Col-0 seedlings with TRIzol (RNAiso plus, 9109). First-strand cDNA was synthesized with an 18-mer oligo (dT) primer. Subsequent PCR amplification of *AtTPPF* cDNA involved the primer pair TPPF-F and TPPF-R (Additional file 5: Table S1).

The PCR products were fused to the Cauliflower mosaic virus (*CaMV*) *35S* promoter, which was then inserted into a *pMDC140* binary vector with Gateway Technology (Invitrogen). The constructs were introduced into *Agrobacterium tumefaciens* strain *GV3101* and transformed via floral infiltration into Col-0 (for overexpression analysis) and *tppf1* mutants (for complementation experiment).

### RT-PCR and RT-qPCR analysis

Total RNA was isolated from two-week-old seedlings using TRIzol (Takara, RNAiso plus, 9109). First-strand cDNA was synthesized. The volume of each cDNA pool was adjusted to give the same PCR signal strength for *eIf-4A* after 28 cycles. The RT-PCR products were analyzed by electrophoresis on a 1.0% agarose gel. All PCRs were performed in triplicate.

RT-qPCR analysis was performed with a SYBR Premix Ex Taq Kit (TaKaRa) using a CFX96 Real-time PCR Detection System (Bio-Rad), and *eIF-4A* was used as the internal standard. The RT-qPCR primers are given in Additional file 6: Table S2.

### Estimation of trehalose contents

Trehalose content is estimation in accordance with the manufacturer’s protocol (Solarbio, BC0330). The principle of this method is the same as that of the determination of soluble sugar –“Anthrone colorimetry”. The only difference is the application of standard substance. The trehalose (10 mg/mL) was used for the drawing of standard curve in trehalose content measurements. However, this method has some limitations and only could estimate trehalose content because the signal produced by other soluble sugars in plants might affect the determination. Two-week-old seedlings grown on MS medium were treated with or without 20% polyethylene glycol (PEG) for 4 h, approximately 0.1 g leaf tissue was sampled to determine the trehalose content. The sample was ground in liquid nitrogen and 1 mL extraction buffer was added for homogenization. After incubating for 45 min at room temperature (the homogenate was mixed 3–5 times during the incubation period), the sample was centrifuged at 8000×*g* at 25 °C for 10 min. The supernatant was used for the determination of trehalose content. First, draw the standard curve with the standard trehalose solution (10 mg/mL). Mix 0.25 mL trehalose standard liquid of different concentration with 1 mL anthrone dissolved in 80% H_2_SO_4_ and then keep in water bath at 95 °C for 10 min, and the absorbance of the mixture was recorded at 620 nm. The primed sample supernatant was determined by the same method. Trehalose content is calculated from a standard curve of trehalose at 620 nm.

### Analysis of H_2_O_2_ accumulation

H_2_O_2_ accumulation was measured using DAB staining. For DAB staining assays, seedlings grown on MS plates for 10 d were left untreated or treated with PEG6000 for 24 h and then incubated in staining buffer (0.1 mg/mL DAB dissolved in 0.1 M HCl, pH 3.8). After 12 h, seedlings were distained in 75% ethanol for subsequent microscopy.

### Soluble sugar content measurement

The total soluble sugar measurements was performed as described as the reference [39]. Ten-day-old seedlings on a plastic slide were treated with or without 20% PEG for 1 d, after which approximately 0.1 g of leaf tissue was sampled for the determine of the soluble sugar. The sample of 0.1 g was collected and homogenization in 2.5 mL ddH_2_O. Boiled for 15 min, and after cooled at room temperature, centrifuged at 12,000 rpm for 15 min. Then the supernatant was used for the determination. Mix 50 μL supernatant with the 2.5 mL anthrone regent and boiled them for 10 min, cooled at room temperature and then the absorbance of the mixture was recorded at 620 nm. The content of soluble sugar content is calculated from a standard curve of glucose at 620 nm.

### Sucrose content measurement

Sucrose content was determined by the plant tissue sucrose content detection kit (Solarbio, BC2465). Ten-day-old seedlings on a plastic slide were treated with or without 20% PEG for 1 d, after which approximately 0.1 g of leaf tissue was sampled for the sucrose measurements. After the sample was grounded to powder, 0.5 mL extraction buffer was added and incubated it at 80 °C for 10 min. After which, the sample was destained and centrifuged at 4000×g at 25 °C for 10 min. The fundamental method was resorcinol method and the protocol was similar with the reference [40]. 50 μL of the aforementioned supernatant was added into 50 μL 2 M NaOH, boiled for 5 min at 100 °C, cooled down at room temperature, then mixed with 350 μL HCl and 100 μL 0.1% resorcinol. Then the mixture was water bathed at 100 °C for 10 min and was cooled down to room temperature. The content of sucrose was measured at A480.

### Yeast one-hybrid assay

The yeast one-hybrid assay was done according to the Matchmaker One-Hybrid System User Manual (Clontech). Plasmid *pJG4–5* was used to express DREB1A to create the prey vector. Reporter plasmid *pLacZi-2 μ* was modified from *pLacZi* (Clontech) as previously described; it is a high copy autonomous vector [41]. To construct the prey vector, the DREB1A coding sequence was amplified by PCR from Phanta Super-Fidelity DNA Polymerase (Vazyme) and cloned into the *pJG4–5* vector through ClonExpress II One Step Cloning Kit (Vazyme, C112–01) using the linearization of pJG4–5 with *EcoRI* and *XhoI* site. Primers with adaptor were DREB1A-F and DREB1A-R (Additional file 5: Table S1). To create the bait vectors *P1-pLaCZi-2 μ*, the *P1* promoter regions containing the DRE/ARE cis-element. *P1* were amplified via PCR with P1-F and P1-R (Additional file 5: Table S1) using genome DNA template of wild-type. The PCR product was introduced into the *pLacZi-2 μ* vector with the ClonExpress II One Step Cloning Kit using the linearization of *pLacZi-2 μ* with *EcoRI* and *KpnI* site. The two construct was co-transformed into the yeast strain *EGY48* through the protocol described in the Matchmaker One-Hybrid System User Manual and selected on the respective selective dropout media (SD) lacking uracil and tryptophan and supplemented with X-Gal. Each interaction was tested three times in parallel in three independent experiments.

### Dual luciferase reporter assays

The full-length DREB1A open reading frame (ORF) was amplified from WT cDNA via the gene-specific primers DREB1A-F1 and DREB1A-R1 (Additional file 5: Table S1) and cloned into the effector vector *pGreenII62-SK* [42] under the control of the *CaMV 35S*. A 539-bp *AtTPPF P1* promoter fragment was amplified with the specific primers P1-F1 and P1-R1 (Additional file 5: Table S1) and then ligated into the reporter vector, *pGreen II 0800-LUC* [42].

The effector and reporter constructs were transformed into *GV3101* via the helper plasmid *pSoup-P19*. *Agrobacterium tumefaciens GV3101* harboring the above plasmids were then infiltrated into tobacco plants in the following combinations and ratios: *LUC + 35S:DREB1A*(1:9); *P1*:*LUC + 62-SK* (1:9); *P1:LUC*+ *35S:DREB1A* (1:9); The infiltrated tobacco plants were grown for an additional 3 d in a growth chamber under a 16-h light/8-h dark photoperiod at 21 °C. Afterward, 2-cm-diameter leaf discs were harvested and ground for luciferase and Renilla assays via the Dual-Luciferase Reporter Assay System (Promega) with an Infinite200 Pro microplate reader (Tecan). The promoter activity was expressed as the ratio of LUC to REN.

### RNA sequencing (RNA-seq) and analysis

Total RNA was extracted from two-week-old seedlings using RNeasy Plant Mini Kit (QIAGEN 74904). Two micrograms of RNA were used for library construction; each sample was replicated three times. The transcriptome data set used in this study was obtained using the Illumina HiSeq platform, and 150-bp high-quality trimmed paired-end reads were generated. The trimmed reads were mapped to the reference genome sequence of Arabidopsis using HISAT2 (http://ccb.jhu.edu/software/hisat2/faq.shtml), with the default settings [26]. Differentially expressed genes were analyzed using edgeR (http://bioinf.wehi.edu.au/edgeR/) [43]. Genes were considered differentially expressed if their change in expression was more than twofold that of WT (*P* < 0.05).

## Additional files

Additional file 1: **Figure S1.** Phylogenetic analysis of the *TPP* genes from Arabidopsis and Rice. The deduced full-length amino acid sequences of 10 members in Arabidopsis, and 10 members in Rice TPP proteins respectively were aligned by MUSCLE 3.8 and the phylogenetic tree was constructed using MEGA 7.0 by the Neighbor-Joining (NJ) method with 1000 bootstrap replicates. Each TPP subfamily has been separated and is depicted using different colors. The black circle is the Arabidopsis *AtTPPF*. (TIF 986 kb)

Additional file 2: **Figure S2.** Complementation assays of the *tppf1* mutant. Analysis of the drought-tolerant phenotype of plants of a complementary line Com1. Two-week-old plants growing in the soil were subjected to dehydration by withholding water for 19 d, after which the image shown was taken. Three independent assays were performed, each of which produced similar results. (TIF 7106 kb)

Additional file 3: **Figure S3.** Coexpression model of *AtTPPF* and *AtDREB1A* under abiotic stress conditions. Coexpression model of *AtTPPF* and *AtDREB1A* under abiotic stress conditions developed using the AtGenExpress Visualization Tool (AVT) on TAIR (http://jsp.weigelworld.org/expviz/expviz.jsp?experiment). At4g12430 is *AtTPPF*, and At4g25480 is *AtDREB1A*. The intensity indicates the corresponding expression value. (TIF 5542 kb)

Additional file 4: **Figure S4.** Autonomous activated verification of *AtTPPF-P* and the interaction of AREB1 and *AtTPPF-P1.* Results of Y1H assays and the growth of yeast cells, *AtTPPF-P*: full-length promoter (P) of the *AtTPPF* gene cotransformed with the *pJG4–5* empty vector; *AtTPPF*-P + AREB1: bait full-length promoter (P) of the *AtTPPF* gene cotransformed with prey AREB1; *AtTPPF-P1*: bait P1 cotransformed with the *pJG4–5* empty vector; *AtTPPF*-P1 + AREB1: bait P1 cotransformed with prey AREB1 on selective medium supplemented with X-Gal. (TIF 2662 kb)

Additional file 5: **Table S1.** Primers for vector construction. (DOC 33 kb)

Additional file 6: **Table S2.** Primers for the qRT-PCR. (DOC 44 kb)

Additional file 7: **Table S3.** The information of 318 drought-repressed genes which up-regulated in OE plants (ERG-U). (XLS 59 kb)

Additional file 8: **Table S4.** The information of 207 drought-induced genes which down-regulated in OE plants (DIG-D). (XLS 40 kb)

## Data Availability

The datasets generated and analyzed during the current study are available from the corresponding author on reasonable request.

## Abbreviations

ABA: Abscisic acid
ABRE: ABA-responsive element
CaMV: Cauliflower mosaic virus
GO: Gene Ontology
MAPK: Mitogen-activated protein kinase
ORF: Open reading frame
qPCR: Quantitative PCR
ROS: Reactive oxygen species
RT-PCR: Reverse transcription polymerase chain reaction
SAM: Shoot apical meristem
T6P: Trehalose-6-phosphate
TPP: Trehalose-6-phosphate phosphatase
