# Supplementary figures and images for: Overexpression of the trehalose-6-phosphate phosphatase family gene AtTPPF improves the drought tolerance of Arabidopsis thaliana
Source: BMC Plant Biol. 2019 Sep 2;19:381. doi: 10.1186/s12870-019-1986-5 (PMC6721209; doi:10.1186/s12870-019-1986-5)

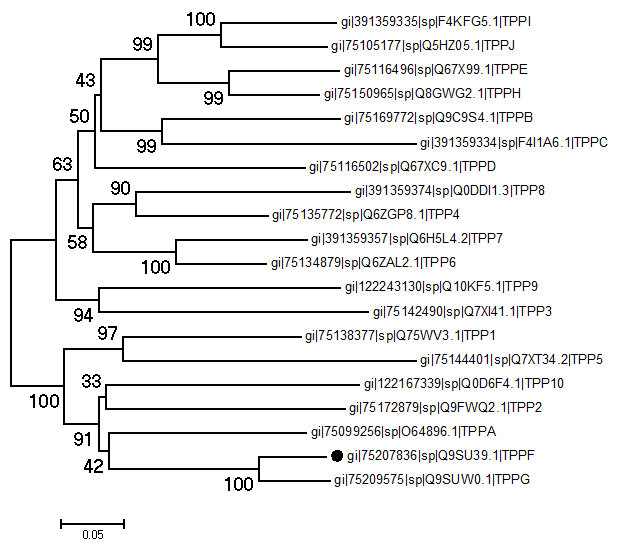

Supplement: Supplementary file 1 — Figure S1. Phylogenetic analysis of the TPP genes from Arabidopsis and Rice. The deduced full-length amino acid sequences of 10 members in Arabidopsis, and 10 members in Rice TPP proteins respectively were aligned by MUSCLE 3.8 and the phylogenetic tree was constructed using MEGA 7.0 by the Neighbor-Joining (NJ) method with 1000 bootstrap replicates. Each TPP subfamily has been separated and is depicted using different colors. The black circle is the Arabidopsis AtTPPF. (TIF 986 kb) [file 12870_2019_1986_MOESM1_ESM.tif]

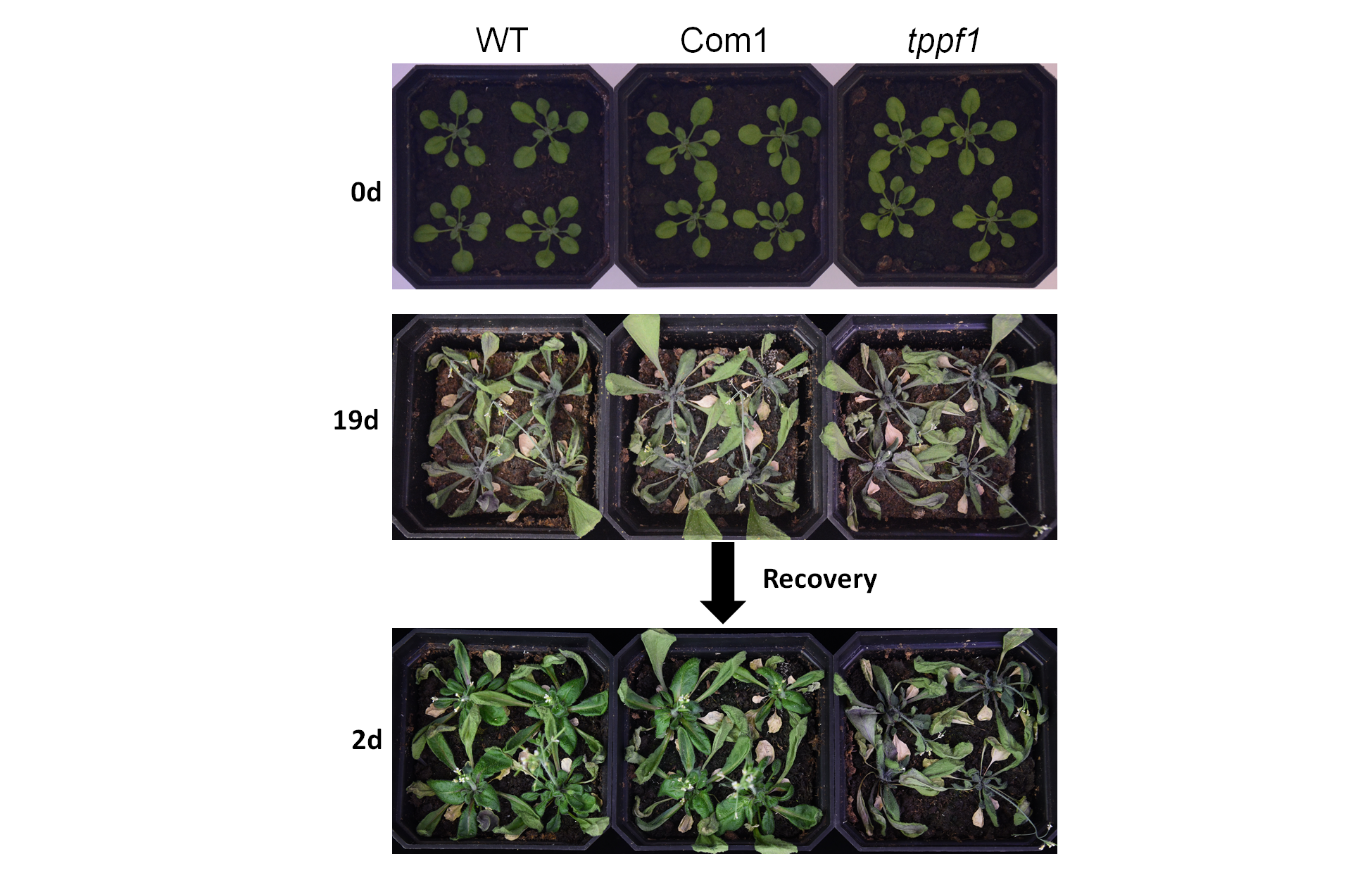

Supplement: Supplementary file 2 — Figure S2. Complementation assays of the tppf1 mutant. Analysis of the drought-tolerant phenotype of plants of a complementary line Com1. Two-week-old plants growing in the soil were subjected to dehydration by withholding water for 19 d, after which the image shown was taken. Three independent assays were performed, each of which produced similar results. (TIF 7106 kb) [file 12870_2019_1986_MOESM2_ESM.tif]

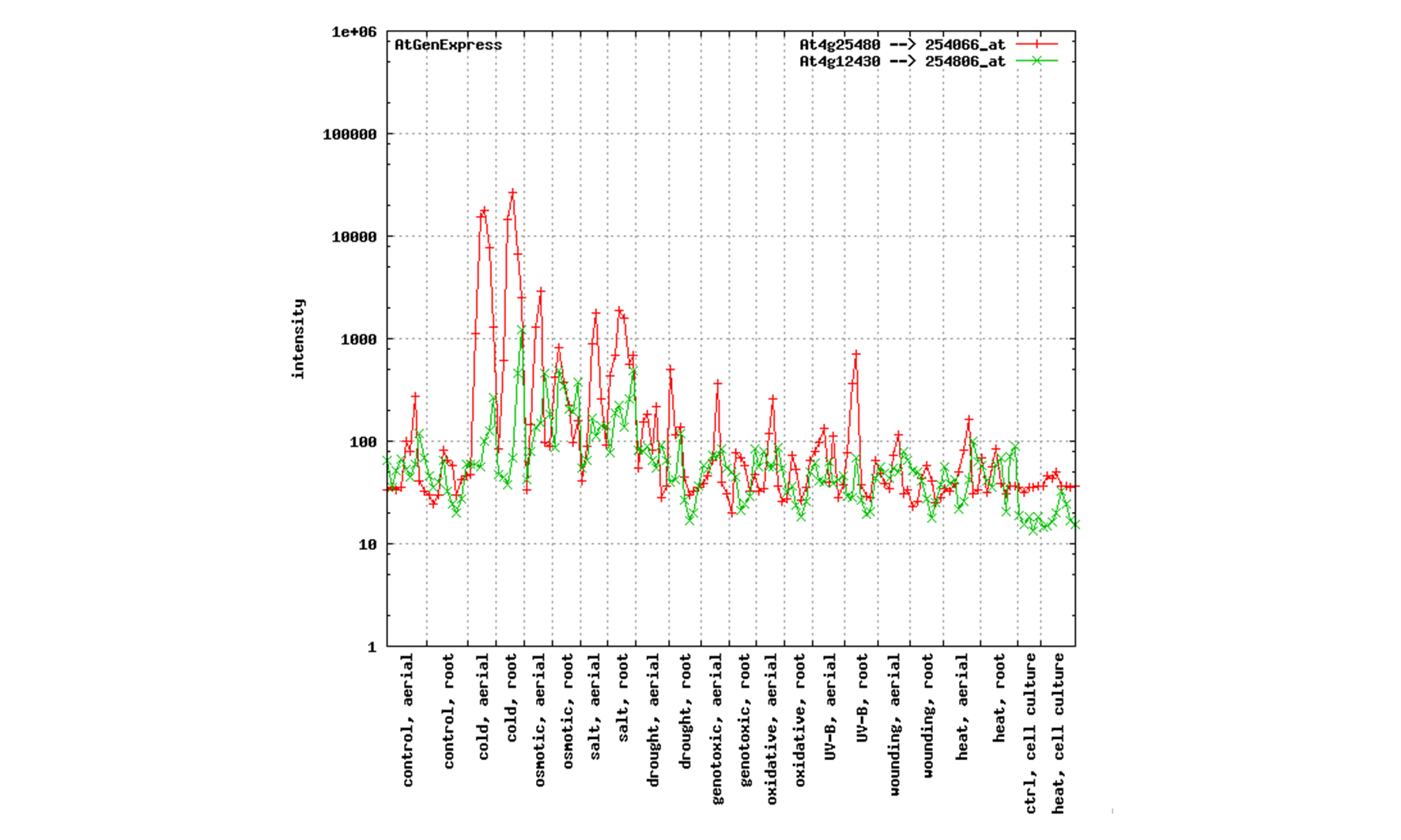

Supplement: Supplementary file 3 — Figure S3. Coexpression model of AtTPPF and AtDREB1A under abiotic stress conditions. Coexpression model of AtTPPF and AtDREB1A under abiotic stress conditions developed using the AtGenExpress Visualization Tool (AVT) on TAIR (http://jsp.weigelworld.org/expviz/expviz.jsp?experiment). At4g12430 is AtTPPF, and At4g25480 is AtDREB1A. The intensity indicates the corresponding expression value. (TIF 5542 kb) [file 12870_2019_1986_MOESM3_ESM.tif]

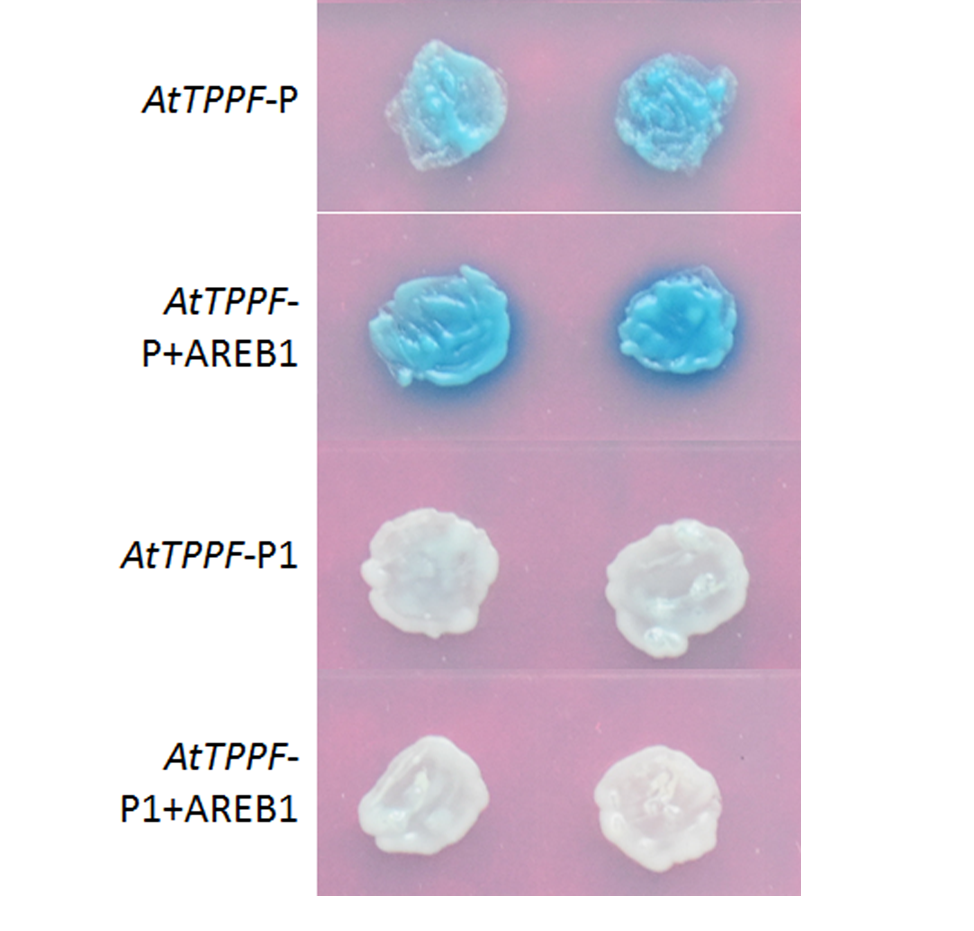

Supplement: Supplementary file 4 — Figure S4. Autonomous activated verification of AtTPPF-P and the interaction of AREB1 and AtTPPF-P1. Results of Y1H assays and the growth of yeast cells, AtTPPF-P: full-length promoter (P) of the AtTPPF gene cotransformed with the pJG4–5 empty vector; AtTPPF-P + AREB1: bait full-length promoter (P) of the AtTPPF gene cotransformed with prey AREB1; AtTPPF-P1: bait P1 cotransformed with the pJG4–5 empty vector; AtTPPF-P1 + AREB1: bait P1 cotransformed with prey AREB1 on selective medium supplemented with X-Gal. (TIF 2662 kb) [file 12870_2019_1986_MOESM4_ESM.tif]
