# Supplementary material for: Overexpression of the trehalose-6-phosphate phosphatase family gene AtTPPF improves the drought tolerance of Arabidopsis thaliana
Source: BMC Plant Biol. 2019 Sep 2;19:381. doi: 10.1186/s12870-019-1986-5 (PMC6721209; doi:10.1186/s12870-019-1986-5)
Supplement: Supplementary file 5 — Table S1. Primers for vector construction. (DOC 33 kb) [file 12870_2019_1986_MOESM5_ESM.doc]

**Table S1: Primers for vector construction**

| **Primer name** | **Primer sequence(5’-3’)** |
| --- | --- |
| AtTPPF-F | GGGGACAAGTTTGTACAAAAAAGCAGGCTTCATGGATTTAAACTCAAACCACA |
| AtTPPF-R | GGGGACCACTTTGTACAAGAAAGCTGGGTCTCAAAAACCAGTAGAATTCTTC |
| DREB1A-F | TATGCCTCTCCCGAATTCATGAACTCATTTTCTGCTTT |
| DREB1A-R | AGTCCAAAGCTTCTCGAGTTAATAACTCCATAACGATACGT |
| P1-F | CCGGAATTCACGTGAATTAACTTCAAAGGCACA |
| P1-R | CGGGGTACCTAGACTCTCTTTTTCTTTTAGA |
| DREB1A-F1 | TATGCCTCTCCCGAATTCATGAACTCATTTTCTGCTTT |
| DREB1A-F1 | AGTCCAAAGCTTCTCGAGTTAATAACTCCATAACGATACGT |
| P1-F1 | CTATAGGGCGAATTGGGTACCACGTGAATTAACTTCAAAGGCACA |
| P1-R1 | TGTTTTTGGCGTCTTCCATGGTAGACTCTCTTTTTCTTTTAGA |
