# Supplementary material for: Overexpression of the trehalose-6-phosphate phosphatase family gene AtTPPF improves the drought tolerance of Arabidopsis thaliana
Source: BMC Plant Biol. 2019 Sep 2;19:381. doi: 10.1186/s12870-019-1986-5 (PMC6721209; doi:10.1186/s12870-019-1986-5)
Supplement: Supplementary file 6 — Table S2. Primers for the qRT-PCR. (DOC 44 kb) [file 12870_2019_1986_MOESM6_ESM.doc]

**Table S2: Primers for** the qRT-PCR

| **Primer name** | **Primer sequence(5’-3’)** |
| --- | --- |
| qTPPF-F | ATGGATTTAAACTCAAACCAC |
| qTPPF-R | TCAAAAACCAGTAGAATTCTTC |
| qDREB1A-F | GATCAGCCTGTCTCAATTTC |
| qDREB1A-R | CTTCTGCCATATTAGCCAAC |
| bHLH038-F | GTTATCTCTCAACGGTTTCTGC |
| bHLH038-R | CCCACCCAACACATTTGATATC |
| bHLH039-F | GGTTTCTCGAAGCTTGAAGTAC |
| bHLH039-R | GCTGCTTAACGTAACATTCAGT |
| MYB90-F | CTTCTTCGCCTTCATAAGCTTC |
| MYB90-R | GGTGTTGTAGGAGGGGAAATAA |
| LTP4-F | CCTTGTTTTAACGGTGTGCATA |
| LTP4-R | ACCAACCCACCTTTTGATAGAT |
| AFP3-F | TGAGTAGGACTTGTTCGTTACC |
| AFP3-R | ACCACCACAAGCTTTATGTTTC |
| COR413IM1-F | GCCTGGATTAAGGGAGAGTATG |
| COR413IM1-R | CTCAAGTTCACCTGGAAAAGTG |
| TPS5-F | TTGGATTATGATGGCACAATGG |
| TPS5-R | TGGTGTTGTCCTAATGGAACCTG |
| DXP-F | AAAGCCGCTTTCCTACAAGGT |
| DXP-R | CAGCGAAGGTAACTGCATGTTG |
| MYB75-F | TCCTAAGGAAAGCCAAGAGGTAGA |
| MYB75-R | AGGTGTCCCCCTTTTCTGTTG |
| TPS8-F | GCACAGTGACAAATCCGGAAC |
| TPS8-R | GCTTGGTTTTCTTCCAACCG |
| TPS9-F | AGTCGTCAAGAGAGGCCAACAC |
| TPS9-R | TAGACCTTTGCTTACTCCCTGTGG |
| LIP-F | TTTGGGCAACCTCGTGTTG |
| LIP-R | ATTGGGCACAAGCAAACTGTAA |
